# Clinical Profile and Outcome of Haemodialysis in Patients With COVID-19 – A Single Centre Experience

**DOI:** 10.7759/cureus.17170

**Published:** 2021-08-14

**Authors:** Harsh Vardhan, Amit Kumar, Shyama Shyama, Neha Chaudhary, Sanjay Pandey, Deependra K Rai, Deepak Kumar, Sanyal Kumar

**Affiliations:** 1 Nephrology, All India Institute of Medical Sciences, Patna, Patna, IND; 2 General Medicine, All India Institute of Medical Sciences, Patna, Patna, IND; 3 Community and Family Medicine, All India Institute of Medical Sciences, Patna, Patna, IND; 4 Physical Medicine & Rehabilitation, All India Institute of Medical Sciences, Patna, Patna, IND; 5 Respiratory Medicine, All India Institute of Medical Sciences, Patna, Patna, IND; 6 Physical Medicine & Rehabilitation, Employees' State Insurance Corporation (ESIC) Medical College and Hospital, Patna, Patna, IND

**Keywords:** acute kidney injury, chronic kidney disease, corona virus, covid-19, haemodialysis, sars-cov-2, transplant

## Abstract

Introduction

Severe acute respiratory syndrome coronavirus 2 (SARS-CoV-2) causing COVID-19 disease is the third coronavirus to have emerged in the last 20 years. The COVID-19 infection causes more severe illness in patients with comorbid diseases, especially in patients with diabetes, hypertension and kidney failure.

Methods

This is a retrospective study using electronic records and laboratory data of adult patients hospitalised at All India Institute of Medical Sciences (AIIMS), Patna between May 1st, 2020 and March 31st, 2021, who were diagnosed with COVID-19 and needed haemodialysis. The demographic characteristics, co-morbidities, symptoms, clinical course, laboratory parameters, and treatments were recorded. The aim of this study is to evaluate the clinical profile and outcome of patients on hemodialysis with COVID-19 infection.

Results

The study included 261 COVID-19 patients who needed haemodialysis. The most common symptoms on admission were fever (72.8%), cough (64.3%) and dyspnoea (46.6%). The mean age was 58.4 +/-15 years. A total of 195 patients (74.7%) were male. The most common co-morbid condition was hypertension (85.1%) followed by diabetes (71.9%). A total of 118 (45.2) patients had acute on chronic kidney disease (CKD), 40 (15.3) were on maintenance haemodialysis (MHD) and 103 (39.5) were having acute kidney injury (AKI). Eight patients were renal transplant recipients. At presentation, 183 (70.1%) patients were having mild to moderately severe infection and 78 (29.9%) patients were having severe disease. A total of 213 patients required ICU admissions, 186 (75.3%) of whom required invasive ventilation. Overall mortality was 66% (172/261) and the rest were discharged.

Conclusion

The study suggests that COVID-19 disease has a significantly more severe course and poorer outcome in patients requiring haemodialysis.

## Introduction

Severe acute respiratory syndrome coronavirus 2 (SARS-CoV-2), the causative agent of COVID-19 disease, was first described in humans in December 2019 in Wuhan, China [1] and is the third coronavirus to have emerged in the last 20 years. Previous outbreaks of the severe acute respiratory syndrome coronavirus (SARS-CoV) in 2002 and the Middle East respiratory syndrome coronavirus in 2012 have been toppled in case incidence by COVID-19 [2] and was declared a pandemic by the World Health Organization. Worldwide 176,945,596 cases have been reported with 3,836,828 deaths till June 18, 2021 [3]. In India, this infection has also caused a significant impact on public health with 2,98,22,411 cases reported till June 18, 2021 with 3,85,164 deaths. The rising cases and its impact on public health forced the Indian government to impose a nationwide lockdown and this impacted the care of patients with kidney disease especially those on maintenance haemodialysis (MHD) [4-6]. The potential impact of SARS-CoV-2 on the kidneys is still undetermined, but emerging evidence indicates that kidney complications are frequent, and COVID-19 disease may have unique features in individuals on chronic dialysis and kidney transplant recipients [4-7].

Our centre was declared a dedicated COVID hospital by the state government. Initially, we were the only dedicated dialysis centre for COVID-19 patients in our state.

## Materials and methods

This is a hospital record-based retrospective observational analytical study conducted at All India Institute of Medical Sciences (AIIMS), Patna focusing on the clinical characteristics and outcome of confirmed cases of COVID-19 who were admitted from May 1st, 2020 to March 31st, 2021 and underwent haemodialysis. The aim of this study is to evaluate the clinical profile and outcome of patients on hemodialysis with COVID-19 infection. This study was approved by the ethics committee of AIIMS Patna (AIIMS/Pat/IRC/2020/672).

Data regarding 261 patients were collected and analysed. A confirmed case with COVID-19 was defined as a positive result to high-throughput sequencing or real-time reverse-transcriptase polymerase-chain-reaction (RT-PCR) assay for nasal and pharyngeal swab specimen. The subjects were followed from admission to death or discharge. Demographic details, medical history, clinical features and laboratory parameters including the inflammatory markers were noted. History elicited regarding smoking habits and co-morbidities like diabetes mellitus (DM), hypertension (HTN), chronic obstructive pulmonary disease (COPD) and coronary artery disease (CAD) were also noted. Renal condition was summarized in three categories: acute kidney injury (AKI), acute on chronic kidney disease and end-stage renal disease (ESRD) on MHD. Other clinical details comprised of history of renal transplant and number of sessions of haemodialysis done during the treatment, oxygen saturation and respiratory rate at the time of admission.

The degree of severity of COVID-19 was defined as mild, moderate and severe according to “National Clinical Management Protocol COVID-19”. AKI was defined according to Kidney Disease: Improving Global Outcomes (KDIGO) guideline as having one of the following: an increase in serum creatinine (Cr) by ≥0.3 mg/dL within 48 h, or an increase in Cr to >1.5 times baseline within the previous seven days, or urine volume <0.5 mL/kg/h for >6 h [8].

## Results

The baseline characteristics of the patients are shown in Table 1.

**Table 1:** Baseline characteristics of patients according to the severity of disease. COPD: Chronic obstructive pulmonary disease; CKD: Chronic kidney disease; MHD: Maintenance haemodialysis; AKI: Acute kidney injury.

Characteristics		Total (N = 261)	Mild/Moderate	Severe	p-value
At presentation					
Age (years) n (%)	< 45 years	43 (16.5)	28 (15.3)	15 (19.2)	0.43
	> 45 years	218 (83.5)	155 (84.7)	63 (80.8)	
Gender n (%)	Female	66 (25.3)	47 (25.7)	19 (24.4)	0.82
	Male	195 (74.7)	136 (74.3)	59 (75.6)	
Current Smoker n (%)		28 (10.7)	21 (11.5)	7 (9)	0.55
Comorbidities n (%)	Diabetes mellitus	187 (71.9)	140 (76.5)	47 (61)	0.01*
	Hypertension	222 (85.1)	155 (84.7)	67 (85.9)	0.8
	COPD	21 (8.1)	15 (8.2)	6 (7.7)	0.89
	Coronary artery disease	60 (23)	45 (24.6)	15 (19.3)	0.35
Renal Syndrome n (%)	Acute on CKD	118 (45.2)	88 (48.1)	30 (38.5)	0.35
	MHD	40 (15.3)	27 (14.8)	13 (16.7)	
	AKI	103 (39.5)	68 (37.2)	35 (44.9)	
Transplant n (%)		8 (3.1)	7 (3.8)	1 (1.3)	0.28
SPO2 at room air Mean (SD)		94.7 (4.7)	97.1 (1.8)	89 (4.5)	<0.001*
Respiratory rate (Per minute) Median (IQR)		29 (26-32)	28 (26-30)	34 (30-40)	<0.001*
During the course of treatment after admission					
Dialysis Median (IQR)		2 (1-4)	3 (1-5)	2 (1-4)	0.03*
Non-Invasive ventilation n (%)		38 (15.6)	31 (17.4)	7 (10.6)	0.19
Invasive ventilation n (%)		186 (75.3)	122 (68.5)	64 (92.8)	<0.001*
Death n (%)		172 (65.9)	113 (61.8)	59 (75.6)	0.03*

A total of 183 (70.1%) patients were having mild/moderate disease at presentation whereas 78 (29.8%) patients were having severe disease. The mean age of patients was 58.4 +/- 15.1 years. The majority (83.5%) were aged more than 45 years and were male (74.7%). The most common co-morbid condition was hypertension (222, 85.1%) followed by diabetes mellitus (187, 71.9%). Twenty-eight (10.7%) patients were smokers at the time of presentation. A total of 118 (45.2%) patients were suffering from acute on chronic kidney disease (CKD) followed by AKI (103, 39.5%) and 40 (15.3%) patients were on MHD. Eight (3.1%) patients were renal transplant recipients. The mean saturation of oxygen at room air at admission (Spo2) was 94.7+/- 4.7. A total of 213 patients required ICU care during the course of treatment and 186 (75.3%) of them required invasive ventilation. Also, the proportion of patients on invasive ventilation was significantly higher among severely ill (92.8%) as compared with mild/moderate (68.5%) [p value<0.001].

Conventional haemodialysis was done in hemodynamically stable patients and sustained low-efficiency dialysis (SLED) in those who were hemodynamically unstable. A total of 143 patients received SLED, 24 at presentation and 119 during the course of treatment while others were managed with conventional haemodialysis. The facility of continuous renal replacement therapy (CRRT) was not available at our centre.

Vascular access was arterio-venous fistula (AVF) in 39 patients and right tunnelled dialysis catheter in one of the patients on MHD. While in patients with acute on CKD and AKI haemodialysis was done through a temporary dialysis catheter.

Indications of dialysis in patients with acute on CKD and AKI were refractory hyperkalaemia, refractory metabolic acidosis, fluid overload and worsening azotemia.

In this study, we did not report any clotting of the dialysis circuit. The baseline laboratory parameters taken at the time of admission of patients are shown in Table 2.

**Table 2:** Baseline laboratory parameter of patients at the time of admission

Laboratory findings	Overall	Mild/Moderate	Severe	p-value
Median (IQR)				
Total leucocyte count (TLC) (μ/L)	13.3 (8.7-19.6)	13 (8.6-19.8)	13.7 (9.4-19.6)	0.55
Absolute lymphocyte count (μ/L)	594.6 (357.4-886)	664.1 (400.7-1122)	567.8 (348.5-848.4)	0.043
Platelet count (μ/L)	135 (98-211)	136 (97-219)	134.5 (98-186)	0.35
Lactate dehydrogenase (LDH) (U/L)	942.5 (648.1-1364)	905.6 (678.3-1284)	1127.1 (654.8-1508.8)	0.17
C-reactive protein (CRP) (mg/L)	109.6 (54.7-210.1)	107.4 (53-204)	121.1 (55.5-225.4)	0.26
D-dimer (mcg/ml)	3 (1.7-7.9)	3.1 (1.7-7.6)	2.87 (1.7-8.5)	0.55
Procalcitonin (mcg/ml)	2.1 (0.7-7.4)	2.1 (0.8-5.8)	2.6 (0.6-9.17)	0.57
Ferritin (ng/ml)	999.9 (533-2132.1)	1181.6 (546-2400)	918.35 (478.5-1900)	0.22
Interleukin 6 (pg/l)	57 (21-169)	53.9 (21.1-144.1)	74.4 (20.6-290.5)	0.12
Creatinine (mg/dl)	4.8 (3.1-7)	4.95 (3.3-7.2)	4.5 (2.9-6.7)	0.30
Mean (SD)				
Albumin (g/dl)	2.7 (0.51)	2.8 (0.5)	2.69 (0.52)	0.07

Except for absolute lymphocyte count (ALC), there was no statistically significant difference with respect to the other different laboratory parameters among the mild/moderate and severely ill patients. The median ALC was significantly lower among severely ill 567.8 (μ/L) (348.5-848.4) as compared with the mild/moderate ill patients 664.1 (μ/L) (400.7-1122) [p-value = 0.043].

Overall, 171 (65.9%) admitted patients did not survive and the mortality rate was significantly higher among severely ill (75.6%) as compared with mild/moderately ill (61.8%) (p-value < 0.03) (Table 3). The proportion of patients aged more than 45 years was higher among those who died (87.8%) as compared with those who survived (75.3%) [p-value = 0.01]. On clinical evaluation, the mean saturation of oxygen at admission was higher 95.8 (3.5) among those who survived as compared with those who did not survive 94 (5) [0.003]. Likewise, a significant difference was observed for respiratory rate i.e., death 30 (28-34) vs. discharge 27 (26-28). A significantly higher proportion of patients who died were suffering from AKI (44.8%) as compared with those who survived (i.e., 29.2%) [p < 0.001]. 9/26 AKI who survived remained dialysis dependant at discharge vs. 33/39 in acute on CKD (Table 3).

**Table 3:** Baseline characteristics of patients according to outcome (N = 261) COPD: Chronic obstructive pulmonary disease; CKD: Chronic kidney disease; MHD: Maintenance haemodialysis; AKI: Acute kidney injury.

Characteristics		Discharge N (%)	Death N (%)	p-value
At presentation				
Age (years)	< 45 years	22 (24.7)	21 (12.2)	0.01*
	> 45 years	67 (75.3)	151 (87.8)	
Gender	Male	23 (25.8)	43 (25)	0.88
	Female	66 (74.2)	129 (75)	
Current Smoker		9 (10.1)	19 (11.1)	0.82
Comorbidities	Diabetes mellitus	70 (78.7)	117 (68.4)	0.08
	Hypertension	75 (84.3)	147 (85.5)	0.79
	COPD	8 (9)	13 (7.6)	0.69
	Coronary artery disease	19 (78.7)	41 (76.2)	0.65
Renal Syndrome	Acute on CKD	39 (43.8)	79 (45.9)	<0.001*
	MHD	24 (27)	16 (9.3)	
	AKI	26 (29.2)	77 (44.8)	
Kidney Transplant recipients		3 (3.4)	5 (2.9)	0.84
SPO2 at Room air Mean (SD)		95.8 (3.5)	94 (5)	0.003*
Respiratory rate (Per minute) Median (IQR)		27 (26-28)	30 (28-34)	<0.001*
During the course of treatment after admission				
Dialysis Median (IQR)		4 (2-7)	2 (1-3)	<0.001*
Non-Invasive ventilation n (%)		34 (43)	4 (2.4)	<0.001*
Invasive ventilation n (%)		19 (25)	167 (97.7)	<0.001*

A total of 143 patients received SLED; 24 at presentation and 119 during the course of treatment. Amongst the patients who received SLED, 136 died as they were critically ill. Invasive ventilation was found to be significantly associated with mortality as the majority of patients (97.7%) who died were shifted on invasive ventilation after the admission.

A significant difference for the median or mean values of the different laboratory parameters was noted between the survivors and the non-survivors. Thus, the majority of patients who died had deranged leucocyte count (76.7%) and lymphopenia (97.1%). Similarly, majority had hypoalbuminemia (95.4%), raised lactate dehydrogenase (LDH) (93.6%), C-reactive protein (CRP) (98.8%), D-dimer (98.3%), serum ferritin (92.4%) and interleukin 6 level (95.4%). The association of lymphopenia, hypoalbuminemia and the other mentioned deranged laboratory parameters was found to be significantly associated with mortality of patients (p < 0.05) (Table 4).

**Table 4:** Baseline laboratory parameter of patients according to outcome (N = 261) TLC: Total leucocyte count; LDH: Lactate dehydrogenase; CRP: C-reactive protein.

Characteristics			Discharge	Death	p-value
TLC	N (%)	Deranged	45 (50.6)	132 (76.7)	<0.001*
		Normal	44 (49.4)	40 (23.3)	
	Median (IQR)		9.62 (6.9-13.3)	16.3 (10.3-22.2)	<0.001*
Absolute lymphocyte count	N (%)	Decreased	77 (86.5)	167 (97.1)	0.001*
		Normal	12 (13.5)	5 (2.9)	
	Median (IQR)		647.2 (389.1-942.8)	556.6 (327.9-869.4)	0.088
Platelet count	N (%)	Decreased	45 (50.6)	99 (57.6)	0.1
		Normal	44 (49.4)	73 (42.4)	
	Median (IQR)		150 (109-216)	127 (90-211)	0.04*
Albumin	N (%)	Decreased	73 (82)	164 (95.4)	<0.001*
		Normal	16 (18)	8 (5)	
	Mean (SD)		3 (0.5)	2.7 (0.5)	<0.001*
LDH	N (%)	Deranged	77 (86.5)	161 (93.6)	0.06
		Normal	12 (13.5)	11 (6.4)	
	Median (IQR)		785 (552.7-1130.5)	1083.6 (770.2-1506.9)	<0.001*
CRP	N (%)	Deranged	85 (95.5)	170 (98.8)	0.09
		Normal	4 (4.5)	2 (1.2)	
	Median (IQR)		66.5 (33.4-141)	144 (77.5-231.3)	<0.001*
D-dimer	N (%)	Deranged	85 (95.5)	169 (98.3)	0.2
		Normal	4 (4.5)	3 (1.7)	
	Median (IQR)		2.5 (1.6-5)	3.5 (1.8-9.7)	0.02*
Procalcitonin	N (%)	Deranged	71 (79.8)	142 (82.6)	0.6
		Normal	18 (20.2)	30 (17.4)	
	Median (IQR)		1.6 (0.6-3.4)	2.6 (8-9)	0.03*
Serum Ferritin	n %	Deranged	75 (84.3)	159 (92.4)	0.04*
		Normal	14 (15.7)	13 (7.6)	
	Median (IQR)		754 (424.6-1700)	1382.1 (600.9-2250)	0.01*
Interleukin 6	N (%)	Deranged	75 (84.3)	164 (95.4)	0.002*
		Normal	14 (15.7)	8 (4.6)	
	Median (IQR)		21.2 (11.8-86.4)	86.3 (28.7-215.7)	<0.001*
Creatinine	N (%)	Deranged	86 (96.6)	167 (97.1)	0.83
		Normal	3 (3.4)	5 (2.9)	
	Median (IQR)		5.6 (3.4-7.4)	4.6 (2.9-6.7)	0.03*

**Figure 1:**
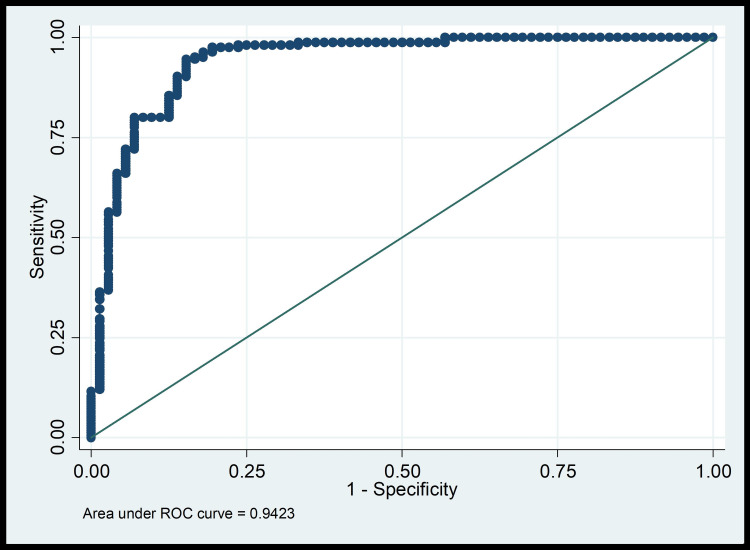
ROC curve of the predictive logistic model

However, no association was noted for procalcitonin and creatinine. Although, median values for both the parameters were significantly higher among the non-survivors. On applying multiple logistic regression, invasive ventilation, deranged total leucocyte count (TLC) and hypalbuminaemia were identified as independent predictors of mortality among COVID patients undergoing haemolysis at the institute. The regression model explained 60.4% variability in patient mortality. Furthermore, the area under receiver operating characteristic (ROC) curve plotted for the regression model was found to be 0.942 which indicates acceptable discrimination for the model (Figure 1).

## Discussion

Our hospital is a tertiary care centre in the state of Bihar in India and it was declared a dedicated hospital for patients with COVID-19 infection. For the initial few months, we were the only government facility providing dialysis services to these patients in our state.

Patients on MHD were the most disadvantageous cohort, as accesses to health care facility were limited especially during the lockdown period [5]. There have been few reports providing the outcome of MHD patients but none so far looked into the outcome of all patients including MHD, AKI and acute on CKD who underwent haemodialysis [9-18].

In this study, 261 patients were included, who underwent haemodialysis in our center, and the study included patients on MHD, AKI and Acute on CKD. The most common symptoms at presentation were fever and/or cough as seen in other studies [9-18].

The hospitalised patients who require haemodialysis need additional manpower and logistics which puts more burden on the already overstretched health care facilities, especially in a developing country like ours. Increased requirement of haemodialysis in critically ill COVID-19 patients leads to a shortage of dialysis machines and other accessories [19].

In our study, the prevalence of CKD was 158 (60.5%) out of which 40 were on MHD. Overall mortality in this group was 115 (73%). The incidence of known CKD patients varied from 0.7-47.6%, depending upon the series described [20, 21]. MHD patients are at increased risk of developing COVID-19 infections as compared to the general population with prevalence ranging from 3 to 9% [14-18]. Sixteen (40%) out of 40 MHD patients succumbed to the illness while mortality in other studies ranged from 19-40% [8-18]. Higher mortality in this study was attributable to a more rapid and severe course of illness.

Patients with kidney diseases had more severe COVID-19 infection with higher mortality as compared to non-CKD patients [21-26]. In this study, 118 patients had AKI with underlying CKD, out of which 88 (74.6%) patients died. Among survivors also, 33 remained dialysis-dependant at the time of discharge. The incidence of AKI was higher in patients with established CKD [25].

In this study, 103 patients had AKI who underwent haemodialysis, out of which 77 (74.7%) patients died. Most of these patients required ICU care and invasive ventilation. Silver et al. in their systematic review and meta-analysis reported a pooled prevalence of AKI as 28% in non-ICU setting and 46% in ICU setting in patients with COVID-19 infection. The pooled prevalence of renal replacement therapy (RRT) was 9% in non-ICU setting and 19% in ICU setting [25].

Mortality in AKI ranged from 1% to 61% in various studies and depends on the study population and mortality amongst those requiring RRT ranged from 45% to 80% [25, 26]. Xu et al. reported a 60-day mortality of around 90% in AKI requiring RRT in critically ill patients [26]. Among survivors in AKI cohort, nine were dialysis-dependant at discharge in this study.

No clotting of the dialysis circuit was reported in this study with routine anticoagulation with un-fractionated heparin. While other studies report a high incidence of clotting mainly during CRRT [27]. The pathogenesis of high thrombosis in COVID-19 infection is incompletely understood but endothelial inflammation, hyper viscosity and pulmonary hypoxemia leading to vasoconstriction may contribute [28].

A significantly higher proportion of patients who died were suffering from AKI (44.8%) as compared with those who survived (i.e., 29.2%) [<0.001]. On applying multiple logistic regression need for invasive ventilation, raised TLC and hypalbuminaemia were identified as independent predictors of mortality.

Age more than 45 years, the mean saturation of oxygen at presentation, higher respiratory rate at presentation and the requirement for invasive ventilation were found to be significantly associated with mortality. Also, the majority of patients who died had raised leucocyte count (76.7%), lymphopenia (97.1%), hypoalbuminemia (95.4%), raised LDH (93.6%), raised CRP (98.8%), D-dimer (98.3%), serum ferritin (92.4%) and interleukin 6 level (95.4%). While raised leucocyte count, lymphopenia, hypoalbuminemia, raised serum ferritin and raised IL-6 were found to be significantly associated with mortality of patients. In the study by Wu et al., older age, higher Sequential Organ Failure Assessment (SOFA) score and D-Dimer greater than 1 µg/mL on admission were associated with mortality [29].

Shang et al. in their study reported certain laboratory parameters including CRP, neutrophil count, LDH, white blood cell count, albumin, and procalcitonin predictive of the prognosis of MHD patients with COVID-19 with CRP being the strongest single predictive indicator [30].

Limitation

The main limitation of this study is its design; being a single-center retrospective study. Final outcome of patients who were discharged is not available. The outcome of this cohort was not compared with patients without renal dysfunction or with patients not requiring dialysis. We need a larger multicentric study looking at the outcomes of haemodialysis patients so that it can guide the policy makers for better health care delivery in patients with kidney disease especially in a resource-limited setting.

## Conclusions

Our study suggests that COVID-19 patients who needed hemodialysis had more severe course and had the worst outcome. Patients with AKI had higher mortality as they had more severe disease and required ICU care. Need for invasive ventilation, raised TLC and hypalbuminaemia were independent predictors of mortality. Age, mean oxygen saturation at presentation and raised inflammatory markers were significantly associated with mortality. All efforts should be made to search for preventable causes of AKI. Clinicians should be aware of the potential risk factors of poor outcomes in hemodialysis patients.
